# Morel-lavallee lesion in children

**DOI:** 10.1186/1749-7922-8-60

**Published:** 2013-12-30

**Authors:** Eun Young Rha, Dae Ho Kim, Ho Kwon, Sung-No Jung

**Affiliations:** 1 Department of Plastic and Reconstructive Surgery, Yeouido St. Mary's Hospital, Catholic University of Korea, 62 Yeouido-Dong, Yeoungdeungpo-Gu, Seoul 150-713, Republic of Korea; 2 Department of Plastic and Reconstructive Surgery, Uijeongbu St. Mary’s Hospital, Catholic University of Korea, 271 Cheonbo-ro, Uijeongbu-si, Gyeonggi-do 480-717, Republic of Korea

**Keywords:** Morel-Lavallee lesion, Closed degloving injury, Children

## Abstract

Morel-Lavallee lesion (MLL) is a closed degloving injury resulting from blunt shearing or tangential forces. In this condition, hemolymph is collected in the closed space between the separated subcutaneous tissue and the underlying fascia. The clinical manifestation of MLL varies from soft fluctuant swelling to skin necrosis or wound sepsis. Due to its inconsistent clinical manifestations and delayed onset, it is rarely described. We present a case of a 28-month-old child who developed delayed MLL arising from pelvic fracture after a motor vehicle accident. In addition, we provide a review of MLL and describe rare cases of it in children.

## Introduction

Morel-Lavallee lesion (MLL) is a closed, soft-tissue degloving injury that is accompanied by disruption of perforating vessels and lymphatics. It occurs as a result of blunt shearing or tangential forces that separate the mobile subcutaneous tissue from the immobile underlying fascia. In this disorder, hemolymphatic collection is formed in the closed space between the two detached layers [1,2]. The diagnosis of MLL is routinely made based on clinical and radiological examination [3,4]. In 1/3 of cases, there is a possibility that clinicians might fail to diagnose MLL due to its inconsistent clinical manifestations and because it often involves initial skin bruising due to underlying soft tissue injury [2,5-7].

We present a case of delayed MLL arising from pelvic fracture caused by a motor vehicle accident. Based on the available literature, this case involves the youngest individual yet reported to suffer from delayed MLL. In addition, we provide a review of MLL and describe rare cases of the disorder in children.

### Presentation

**Figure 1:**
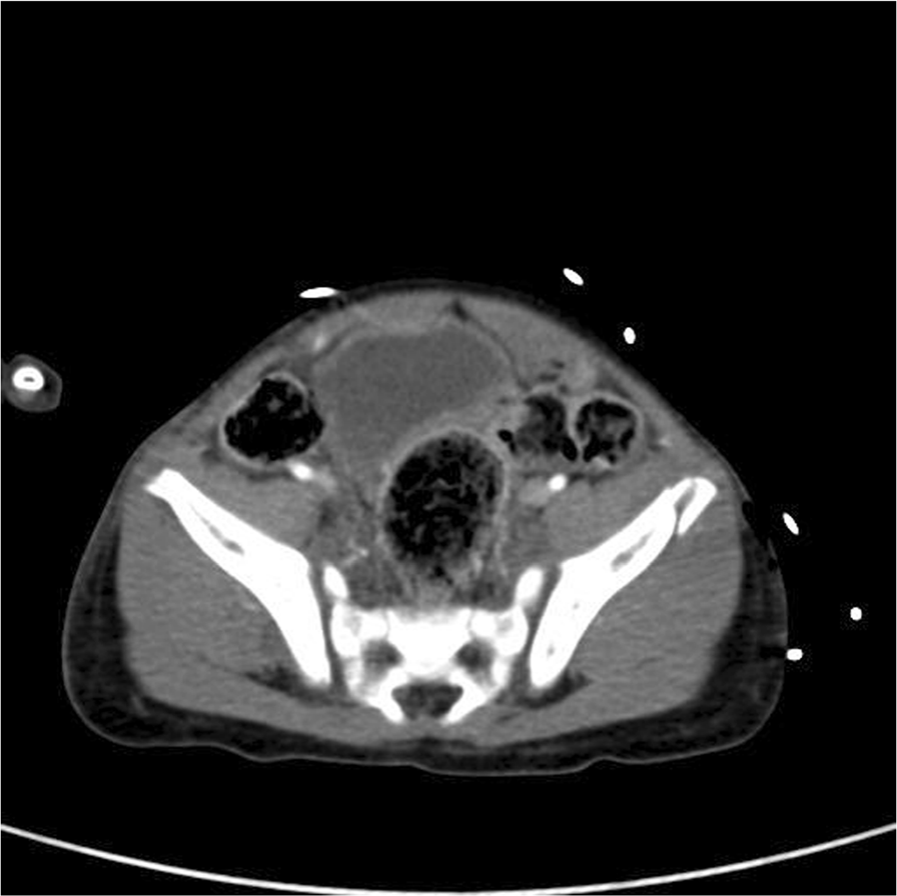
**Initial contrast-enhanced axial CT scan.** The scan shows multiple fractures of the pelvic bone and the hematoma formed in the paravesical and prevesical retroperitoneum.

**Figure 2:**
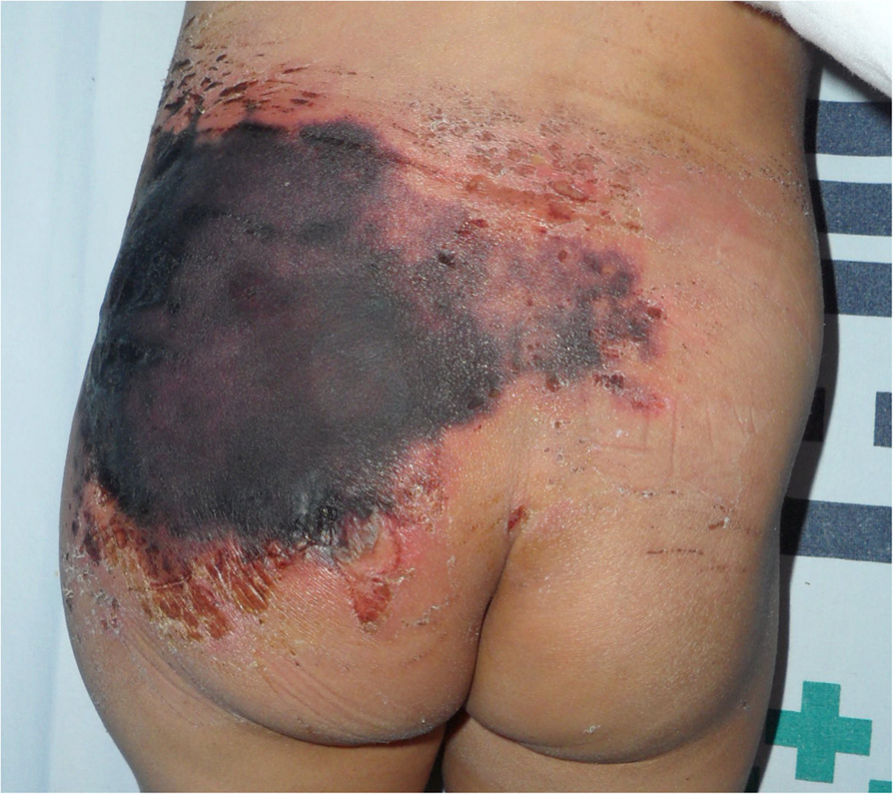
**Clinical image obtained on day 4.** Skin necrosis with black-colored eschar was noted in the left gluteal region.

**Figure 3:**
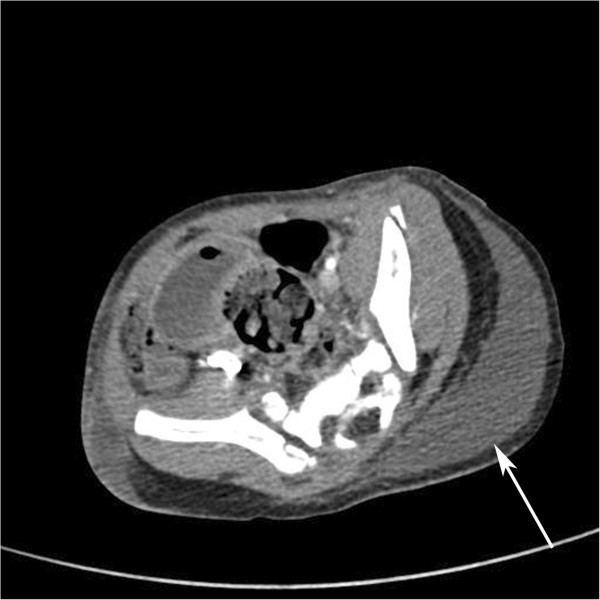
**Contrast-enhanced axial CT scan obtained on day 9.** The scan shows a well-defined isodense to hypodense fluid collection (arrows).

**Figure 4:**
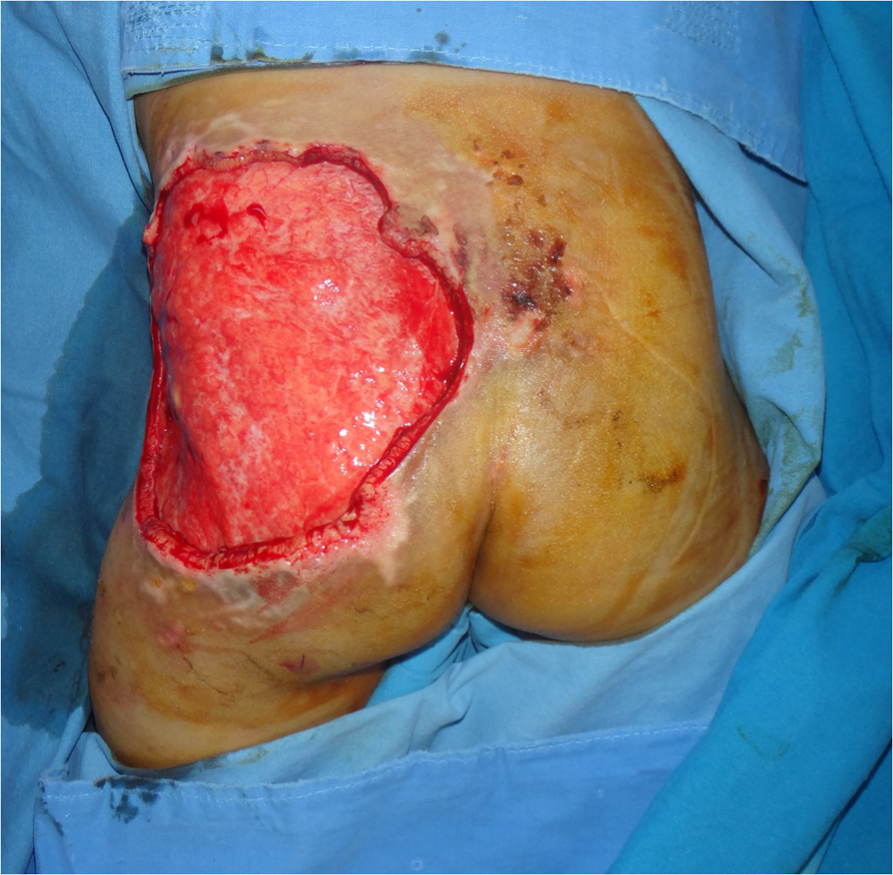
**Clinical image obtained on day 13 after debridement.** A wide skin defect area, including a subcutaneous pocket along the margin of the surrounding skin, was noted.

**Figure 5:**
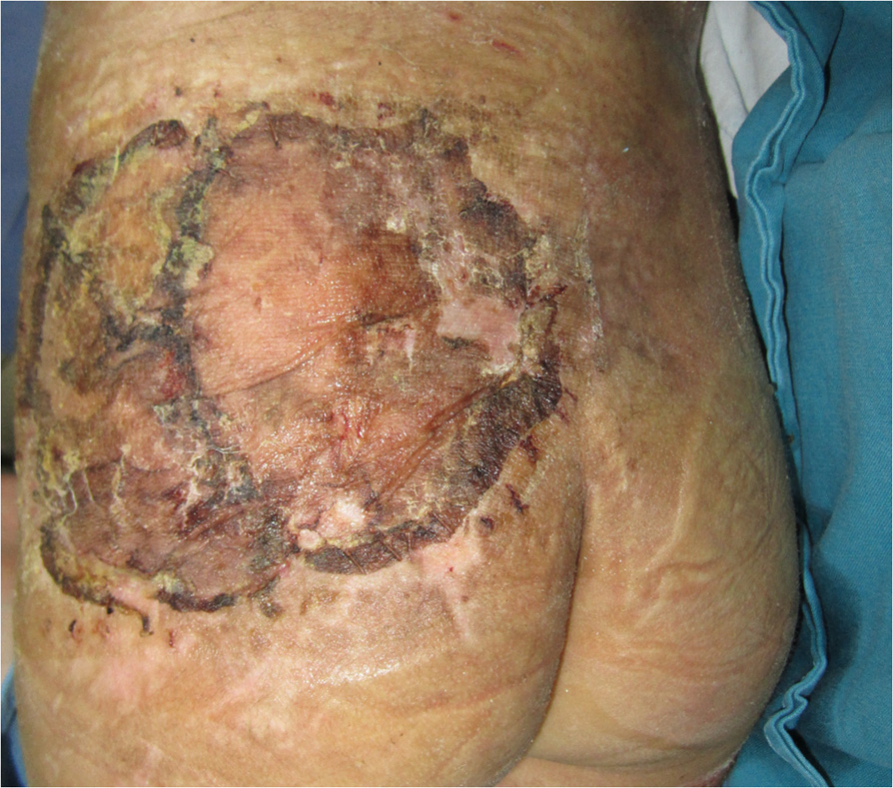
**Postoperative 1-month image.** The skin graft was well taken without any complications.

A 28-month-old child was presented to the department of emergency medicine of our medical institution following a traffic accident. The patient had no history of neonatal injury or developmental abnormality and had stable vital signs and no neurologic symptoms. On physical examination, the patient had mild swelling and tenderness in the right inguinal region but no tenderness or rebound tenderness in the abdomen. Moreover, the patient had a perineal laceration and slight bleeding. The range of motion (ROM) of both hip and knee joints was within the normal range. Initial laboratory examination showed a hemoglobin level of 11.7 and a hematocrit of 35.1. Initial radiographs revealed the presence of a fracture of the left anterior superior iliac spine as well as fractures of the right superior and inferior pubic rami. Computed tomography (CT) scans showed that the patient had a hematoma in the paravesical, prevesical retroperitoneum and subcutaneous emphysema in the left pelvic region (Figure 1). The patient received conservative management, including absolute bed rest and pain control, at the department of orthopedic surgery of our medical institution. On day 3, the patient’s hemoglobin and hematocrit levels had decreased to 6.8 and 20.2, respectively. In addition, the patient showed an increase in the amount of retroperitoneal hematoma on follow-up CT scans. Although this finding might have been due to preexisting pelvic fractures, the patient showed no other internal organ damage and continually received conservative management after transfusion with 2 pints of packed red blood cells (RBCs). On day 4, the patient exhibited darkish skin color changes and necrosis in the left gluteal region (Figure 2). At this point, the patient was referred to us for further evaluation and treatment. The patient was suspected of having MLL, for which we followed conservative management with silvadene occlusive dressing until a demarcation of necrotic skin was achieved. On day 9, although the patient showed a decrease in the amount of retroperitoneal hematoma on follow-up CT scans, hematoma or fluid collection was identified in the space between the subcutaneous area and the fascia. Based on these findings, we established a diagnosis of MLL in our patient (Figure 3). On day 10, the patient displayed a necrotic skin demarcation indicating the boundary between the necrotic and viable areas. The patient underwent partial escharectomy, which resulted in natural drainage of the subcutaneous fluid. The fluid was serous and did not show any signs of infection. On day 13, the patient underwent debridement of a thick eschar 12 × 10 cm in size (Figure 4) under general anesthesia accompanied by the application of a vacuum-assisted closure (VAC) device for the purpose of promoting the growth of healthy granulation tissue. These maneuvers were repeated three times until day 23. Thus, the patient achieved resolution of the pocket under the wound margin as well as formation of healthy granulation tissue. On day 24, the patient underwent a split-thickness skin graft (STSG), through which successful coverage of the skin defect was achieved. At 6-month follow-up, the patient displayed complete cure of the wound without recurrence of fluid collection (Figure 5). In addition, the patient also achieved radiologic union of the pelvic bone and was able to resume daily activities.

## Discussion

MLL was first reported in 1863 by the French physician Maurice Morel-Lavallee, who described it as a post-traumatic collection of fluid due to soft tissue injury [8]. MLL was initially used to refer to injuries involving the trochanteric region and proximal thigh. In recent years, however, the term has been used to describe lesions with similar pathophysiology in various anatomical locations, including the hip and thigh [5,6,9]. MLL commonly occurs as a result of peri-pelvic fracture due to high-impact trauma. However, it may also result from a low-velocity crush injury that occurs during sports activities such as football or wrestling [6,9,10]. The clinical features of MLL vary depending on the amount of blood and lymphatic fluid collected at the site of injury and on the time elapsed since the injury. Moreover, MLL may also concurrently present with symptoms such as soft tissue swelling, contour deformity, palpable bulge, skin hypermobility and decreased cutaneous sensation [6,7]. Furthermore, the presence of a soft fluctuant area due to fluid collection is a hallmark of its physical findings [3,4]. The symptoms of MLL are frequently manifested within a few hours or days following the onset of trauma. In up to 1/3 of total cases, however, symptoms may occur several months or years following the onset of injury. This strongly suggests that obtaining a meticulous history of the patient is essential for making an accurate diagnosis of MLL [2,5-7].

A diagnosis of MLL can be established based on imaging studies of the suspected sites and by physical examination. On radiological examination, it is characterized by the presence of a non-specific, non-calcified soft tissue mass [11,12]. On ultrasonography, it is characterized by hyperechoic (blood-predominant) or anechoic (lymph-predominant) fluid collection depending on the age of the lesion and its predominant content. Acute and subacute lesions less than 1 month old show a heterogeneous appearance with irregular margins and lobular shape. In addition, both chronic lesions and lesions older than 18 months show a homogenous appearance with smooth margins and flat or fusiform shape [12,13]. On CT and magnetic resonance imaging (MRI) scans, MLL lesions are well visualized as well-defined encapsulated fluid collections with fluid-fluid levels. On MRI scans, however, the lesions are better visualized with soft-tissue contrast enhancement. Therefore, MRI is a better choice of imaging modality than CT in making a diagnosis of MLL [12,14]. Based on T1- and T2-weighted MRI scans, MLL can be classified into six types. In addition, the age of the blood within the lesion is a key factor in making an accurate diagnosis of MLL [14-16].

Although various strategies for the treatment of MLL have been reported, including the application of compression bandages, percutaneous aspiration and drainage, open debridement and sclerodhesis, there are no established treatment modalities for patients with MLL [4,9,12,16-33]. Conservative management such as compression bandage application, NSAID medication, physiotherapy and absolute bed rest are considered the first-line treatment regimen in patients with acute, small lesions without underlying fractures. Of these, the compression bandage can be used to supplement other treatment options [4,9,12,16,20,22,28]. Percutaneous drainage can be used to manage larger acute lesions that cannot be resolved with a single application of compression bandages. It may also be attempted along with sclerotherapy as a first-line therapy in patients with chronic lesions [17,24,26,31]. Talc sclerotherapy was introduced by Luria et al. [23] in 2007. Since then, various methods of sclerodhesis, including some that involve the use of alcohol and doxycycline, have been reported. Sclerotherapy is performed by injection of sclerosant into the dead space; the sclerosant is allowed to remain for a few minutes, followed by percutaneous drainage. Sclerotherapy can be used as a first-line therapy in patients with acute lesions that are refractory to compression bandages and in patients with chronic lesions [18,23,25]. In patients with chronic lesions, percutaneous drainage may result in recurrent postoperative hematoma or secondary infection [30]. It is therefore mandatory to combine percutaneous drainage with sclerotherapy. In patients with acute lesions with underlying open fractures and in those with chronic lesions with evidence of infection or tissue necrosis due to a local mass effect, open debridement can be attempted as a first-line therapy. Open debridement may also be considered as the final therapy in patients who are refractory to percutaneous drainage with sclerotherapy [19,21,27,29,30,32,33]. Surgical intervention is also indicated in patients with longstanding MLL with pseudocapsule because they are unresponsive to percutaneous drainage and therefore vulnerable to recurrence [11,27,32,33]. The use of synthetic glue and the quilting suture technique after removal of the fibrous capsule have also been reported to prevent fluid collection in the dead space [1,33-36].

Based on a review of the literature, MLL occurs predominantly in patients in their 30s-40s. To our knowledge, only rare cases of MLL occur in children (Table 1). Letts [37] reviewed 16 pediatric cases of degloving injuries and analyzed the causes and sites of injury. This author classified degloving injuries into those involving anatomical degloving (gloving injuries with skin surface disruption) and those involving physiological degloving (degloving injuries with disruption of the underlying skin vasculature without skin surface disruption). Six of the studied patients suffered from physiologic degloving injuries due to train or motor vehicle accidents involving the leg, buttock and back; the mean age of these six patients was 11 years (range, 6–14 years). All six patients, most of whom received defatted skin grafts, had a concurrent anatomical degloving injury. Harma et al. [22] reported five pediatric cases of MLL, of which two were due to automobile crashes. These authors treated a 6-year-old patient with conservative management and a 14-year-old patient with debridement and local flap coverage. In addition, Mukherjee et al. [12] reported a case of MLL in a 14-year-old boy who presented with a soft tissue mass on the right greater trochanter. For this patient, no data were available regarding a possible past history of trauma or the duration of symptoms. Therefore, these authors made a diagnosis of MLL based solely on ultrasonography and MRI scans. They treated the patient with conservative management with elastic compression bandages. Carlson et al. [19] treated 22 patients with MLL, two of whom were pediatric cases, with debridement and dead space closure. Both of the pediatric cases were caused by motor vehicle accident and were treated immediately after the onset of injury. Choudhary et al. [38] reported a case of a 12-year-old boy who presented with thigh swelling and blistering two weeks after sustaining an injury while riding an all-terrain vehicle (ATV). Based on ultrasonography, the patient was diagnosed with MLL and treated with sotradechol foam injection and doxycycline. This patient had no traumatic lesions in the early stage of injury, but gradually presented with symptoms. An imaging study played a key role in making a diagnosis of MLL in this patient. Anakwez et al. [17] reported a case of MLL that occurred following a knee injury caused by falling on asphalt during a football game. The patient presented with pain and bruising of the knee and thigh but had no notable orthopedic symptoms on physical and radiological examination. Two weeks later, however, the patient exhibited localized bruises and blisters and, based on the results of MRI scans, was subsequently diagnosed with MLL. Aspiration was attempted, but drainage was unsuccessful. The patient was managed conservatively with compression dressings and physical therapy. Most recently, Efrimescu et al. [21] reported a case of MLL in a 14-year-old boy. The patient presented with pain and swelling after the onset of blunt trauma to the lower back and was diagnosed with MLL after simple radiography and MRI. This patient was managed with open drainage.

**Table 1:** A summary of reported cases of MLL in children

**Patient**	**Age/sex**	**Etiology**	**Site**	**Duration from injury to development of symptom**	**Symptoms and sign**	**Associated fracture**	**Associated condition**	**Treatment**	**Complication**	**Reference**
1	6/M	Crush under automible	Lateral lumbar	Unknown		Pelvic fracture	Bladder neck rupture	Conservative treatments	(-)	Harma et al. [22]
2	14/M	Crush under automible	Lumbo-sacral	Unknown		Pelvic, femur fracture	Perianal soft tissue injury	Debridement and local flap	Sacral decubitus ulcer	Harma et al. [22]
3	14/M	Unknown	R greater trochanter	Unknown	Swelling, discomfort, soft tissue mass	(-)	(-)	Elastic compression bandage	(-)	Mukherjeee et al. [12]
4	13/M	Motorvehicle collision	R hip	Immediate		L ulnar fracture, R knee subluxation	L knee laceration, L hand degloving injury	Debridement and dead space closure		Carlson et al. [19]
5	13/M	Motorvehicle collision	Presacral	Immediate		R iliac wing, bilateral anterior ramus, femur, R tibia, fibular fracture	L pulmonary contusion	Debridement and dead space closure		Carlson et al. [19]
6	12/M	ATV accident	L thigh	2 wks	Swelling, blister			Aspiration and sclerodesis with Sotradechol foam injection and doxycycline	(-)	Choudhary et al. [38]
7	11/M	Football	L knee	2 wks	Pain, bruise, open blister, nonfluctuant mass			Compressive dressing and physical theraphy	(-)	Anakweze et al. [17]
8	14/M	Blunt trauma	Lumbar area	2 hrs	Voluminous swelling, bruising			Open drainage	(-)	Efrimescu at el. [21]

Abbreviations: *R* right, *L* left, *wks* weeks, *hrs* hours.

We experienced a case of MLL occurring in a 28-month-old patient. To our knowledge, this represents the youngest case of MLL yet reported. In this patient, no data were available concerning a possible past history of shearing injury. The patient had no abrasions or bruises on initial physical examination, and MLL was therefore not considered in the initial diagnosis. For this reason, the patient initially received conservative management only for the pelvic fracture. Moreover, this patient displayed no fluid collection other than the retroperitoneal hematoma detected on CT scans on admission and on day 3. This patient therefore posed a diagnostic challenge. On day 4, the patient presented with skin color change with swelling and fluctuation. This led to the speculation that not only did fluid collection occur as a result of persistent bleeding from the pelvic fracture in the dead space caused by detachment after the onset of initial shearing injury but also that the resulting mass effect led to the occurrence of skin necrosis.

Pediatric cases of MLL are characterized by the relatively high vulnerability of young patients to trauma. It is also noteworthy that the diagnosis of MLL is often delayed in very young patients, for whom history taking regarding shearing injury and the duration of symptoms is often difficult [12,17,22,38]. It is therefore mandatory to carefully examine the patient for external wounds, bruises, swelling, fluctuation, hypermobility of skin and other clues, such as a tire mark, that may be evidence of shearing injury by exposing the patient from head to toe [37]. Even in patients who initially present immediately after the onset of injury with no symptoms, it is necessary to perform a follow-up physical examination and imaging studies. This is essential for the identification of delayed lesion development. When children and adults are subjected to blunt trauma of the same width, children are vulnerable to higher shock per unit area. It can therefore be inferred not only that children are more vulnerable to developing multiple organ damage due to MLL but also that they are at increased risk of developing fractures or deep organ injuries due to the incomplete development of their musculoskeletal systems. Moreover, children have a relative lack of the shock-absorbing function due to the incomplete development of subcutaneous fat [39]. It can therefore be inferred that pediatric cases of MLL might lead to severe degloving injuries. Furthermore, due to their lower volume of blood, children are vulnerable to hypovolemic shock due to bleeding as well as to skin necrosis due to an abrupt mass effect arising from the collection of internal bleeding in the dead space. Such children should be promptly treated immediately after being diagnosed with MLL.

## Conclusions

MLL is a collection of hemolymph resulting from a closed degloving injury. Its diagnosis and treatment are often delayed because it involves internal degloving without surface penetration. Diagnosis of MLL can be made based on clinical and radiological examination. A number of treatment modalities, ranging from conservative management to open debridement, can be attempted for patients with MLL. However, there are no established case-specific treatment regimens for patients with MLL. Although rare, pediatric cases of MLL deserve special attention. This is true not only because MLL in children may pose a diagnostic challenge due to possible difficulties in determining whether there is a past history of shearing injury but also because MLL in children is associated with an increased frequency of fatal complications compared to MLL in adults. Clinicians should therefore include MLL in the differential diagnosis of patients with trauma, even in the absence of a past history of shearing injury. Moreover, clinicians should also perform both physical examinations and imaging studies in establishing a diagnosis of MLL in children.

### Consent

Written informed consent was obtained from the patient for publication of this case report and the accompanying images.

## Competing interests

The authors declare that they have no competing interests.

## Authors’ contributions

All of the authors were involved in the preparation of this manuscript. EYR wrote the manuscript and reviewed the literature. DHK assisted in the surgery and contributed to the literature search. HK participated in the clinical and surgical management of the patient. S-NJ participated in the conception and design of the study and operated on the patient. All of the authors read and approved the final manuscript.
